# Effect of Inhalational Therapy on Buccal Mucosal Cells in Asthmatic Patients: A Cytological Study

**DOI:** 10.5041/RMMJ.10405

**Published:** 2020-10-14

**Authors:** Mohammed Ismail Benazir, Harikrishnan Prasad, Muthusamy Rajmohan, Kenniyan Kumar Srichinthu, Perumal Prema, Loganathan Mahalakshmi, Gopal Shiva Kumar

**Affiliations:** Department of Oral Pathology, KSR Institute of Dental Science and Research, Tiruchengode, Tamil Nadu, India

**Keywords:** Asthma, buccal mucosa, cytomorphometry, exfoliative cytology, side effects

## Abstract

**Objective:**

Inhalational drugs used in treating asthma have several side effects including those on oral tissues. We therefore designed a study to analyze the effects of inhalational drugs on the buccal mucosal cells of the oral cavity.

**Methods:**

Smears were obtained from clinically normal buccal mucosa of 20 randomly selected asthmatic patients who had been under inhalational therapy for at least 6 months. The Papanicolaou-stained smears were then analyzed for average nuclear area, average cytoplasmic area, and average nuclear area:cytoplasmic area ratio for each patient, and the values were compared with those of 10 healthy controls.

**Results:**

A statistically significant decrease in cytoplasmic area (*P*<0.001) was found in asthmatic patients compared to controls. A significant increase in mean nuclear area:cytoplasmic area ratio (*P*<0.001) was noted in asthmatic patients when compared to controls.

**Conclusion:**

Prolonged use of inhalational drugs in patients diagnosed with asthma is associated with changes in oral epithelial cells. There is a need to assess whether these are the direct adverse effects of such drugs and whether they have any long-term impact on oral tissues.

## INTRODUCTION

Asthma is a growing public health problem affecting over 300 million people worldwide. It is characterized by chronic airway inflammation and increased airway hyper-responsiveness.^1^ Beta-2 agonists, bronchodilators, corticosteroids, and sodium cromoglycate are used singly or in combination in an inhaled form for the management of asthma.^2^ Studies have shown that inhaled drugs used in the treatment of chronic respiratory diseases have adverse effects on the oral health and result in xerostomia, dental caries, candidiasis, ulceration, periodontitis, and taste changes.^1^ Therefore, a study was performed to assess whether these inhaled drugs in asthmatics cause any morphometric changes in the exfoliated cells of buccal mucosa.

## METHODS

The study was conducted on 20 patients diagnosed with asthma who had been under inhalational therapy for at least 6 months (study group) and 10 healthy subjects (control group). Institutional Ethical Committee clearance and Institutional Review Board clearance were obtained before commencing the study. Detailed case history was recorded for each patient. Patients with deleterious habits such as tobacco smoking/chewing, betel quid chewing, or alcoholism were excluded from the study. Similarly, patients with other systemic diseases like diabetes mellitus, anemia, hypertension and those under medications other than for asthma and also women who had attained menopause were also excluded from this study.

A wooden spatula was used to obtain the exfoliated epithelial cells from the clinically normal buccal mucosa from each patient by applying moderate pressure in only one direction. The scrapes were smeared onto the center of clean fresh glass slides and spread over a large area, preventing the clumping of cells. The slides were immediately placed in 95% alcohol fixative to ensure proper fixation. The smears were stained by using the Rapid Papanicolaou (Pap) stain (Biolab Diagnostics Limited, Mumbai, Maharashtra, India).

The Pap-stained smears were subjected to cytomorphometric analysis using the ProgRes Capture Pro 2.8 image analysis software with a research microscope (Olympus BX43, Olympus Corporation, Tokyo, Japan). In each smear 50 clearly defined cells with good staining were selected by systematic sampling in a stepwise manner, moving the microscope stage from left to right, and then down and across, in order to avoid measuring the same cells again. Photomicrographs were captured, and the nuclear area (NA) and cytoplasmic area (CA) for each cell was obtained by drawing around the nuclear and cell boundaries using the digitizer cursor. From these values, the mean NA, mean CA, and ratio of mean NA:CA were calculated for each patient. The values obtained for the study and control groups were then compared using Student’s *t* test assuming unequal variances using SPSS software.

## RESULTS

**Figure 1:**
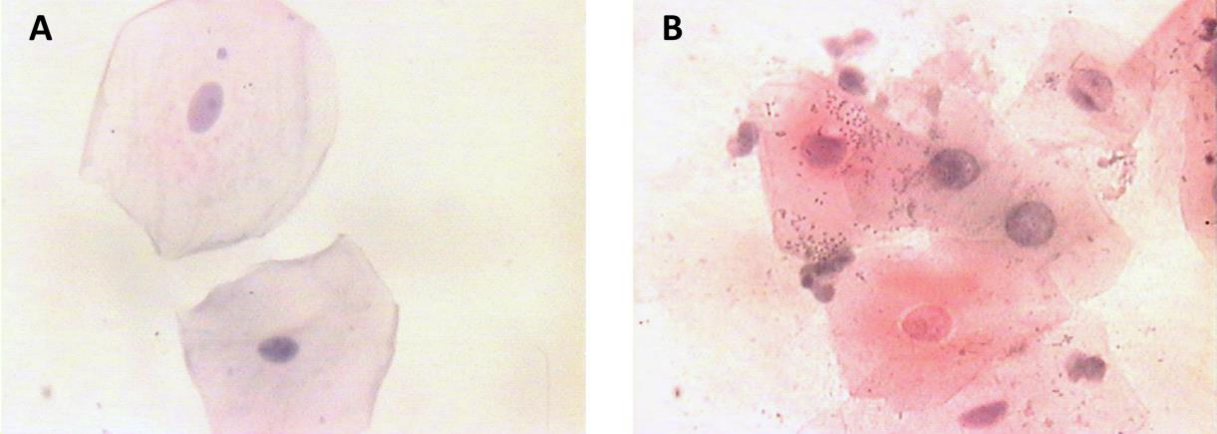
Pap-stained Smear of Control Group (A) and Study Group (B) (100×).

**Figure 2:**
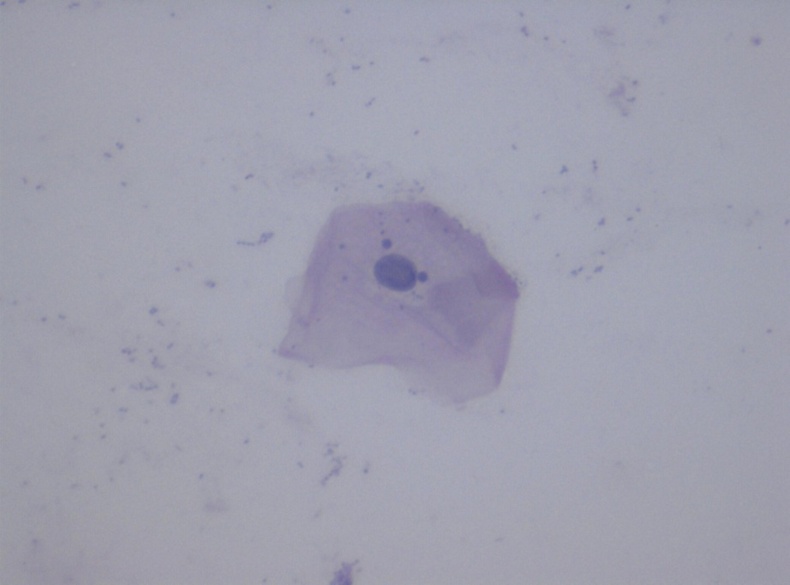
Pap-stained Smear From the Study Group Showing Micronuclei (40×).

**Figure 3:**
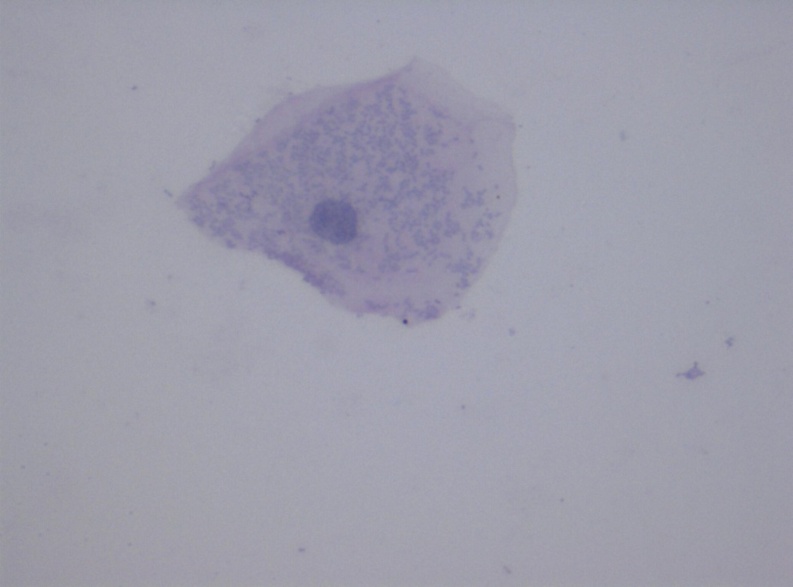
Pap-stained Smear From the Study Group Showing Cytoplasmic Inclusion (40×).

**Figure 4:**
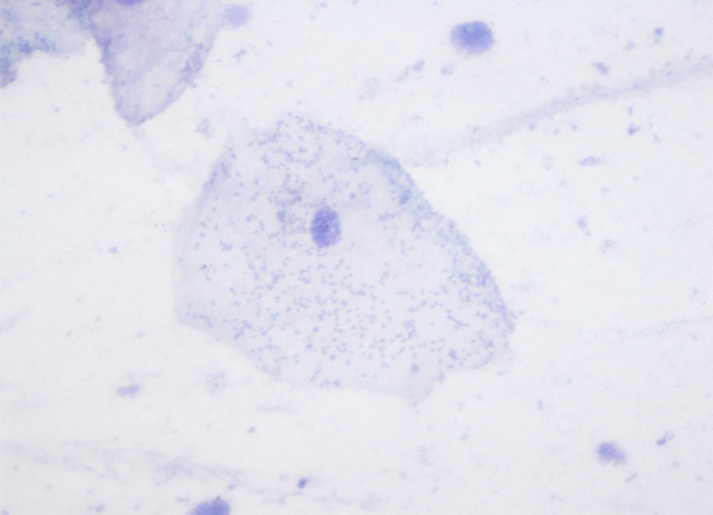
Pap-stained Smear From the Study Group Showing Perinuclear Halo and Karyorrhexis (40×).

Statistical analysis of the data obtained showed that the mean CA was decreased in the study group when compared to the controls (Table 1). The mean cytoplasmic area in the study group was 2310.37 μm^2^ (range: 1713.15 μm^2^ to 2934.16 μm^2^). In the control group, the mean cytoplasmic area was found to be 3187.41 μm^2^ (range: 2970.46 μm^2^ to 3465.52 μm^2^). The difference in the mean CA between the two groups was statistically significant (*P*<0.001). However, the mean NA was slightly higher in the control group when compared to the study group (Table 1; Figure 1). This difference was not significant. The mean NA:CA ratio, as expected, was found to be slightly higher in the study group when compared to the controls (Table 1). This difference was again noticed to be highly significant statistically (*P*<0.001). During the course of microscopic examination of all the smears, we also noticed a greater incidence of morphological changes like micronuclei, cytoplasmic inclusions, karyorrhexis, perinuclear halo, etc. in the smears obtained from the study group (Figures 2–4). However, a quantification of these morphological changes was not done, and hence these were not subjected to any statistical analysis.

**Table 1:** Comparison of Different Parameters between the Study Groups.

Parameter	Study Group Mean (*n*=20)	Control Group Mean (*n*=10)	*t* value	*P* value
Cytoplasmic area (CA) (μm^2^)	2310.37	3187.41	1.705	<0.001*
Nuclear area (NA) (μm^2^)	73.84	77.05	1.701	0.321
NA:CA ratio	0.032	0.023	1.147	<0.001*

* Significant.

## DISCUSSION

Asthma is a chronic inflammatory condition that causes the airways to constrict and produce excess mucus, making breathing difficult. Asthma medications usually consist of bronchodilators and corticosteroids. Most of these drugs are administered using various forms of inhalers or nebulizers. It is well documented in the literature that patients taking asthma medication may be at a higher risk of developing xerostomia, dental caries, dental erosion, periodontal diseases, and oral candidiasis.^1^ In the oral cavity, buccal mucosa is non-keratinized in nature and more vulnerable to change. Therefore, buccal mucosal cells seem to be widely affected by any insult, resulting in cytomorphometric changes.

Many factors affect the morphology of the exfoliated cells collected from the oral mucosa. Some of these are systemic diseases like anemia and diabetes mellitus, or factors such as radiotherapy, alcohol consumption, and smoking. It is for this reason that we did not include such patients in our study. However, to the best of our knowledge, there are no studies assessing the cytomorphometry of oral mucosal cells in asthmatic patients who are under inhalational therapy, apart from a few studies done on sputum specimen. Therefore, we made an attempt to analyze the morphometric and cytological changes in the exfoliated cells of the buccal mucosa in asthmatic patients who have been under inhalational therapy for at least 6 months.

In our study, we measured the nuclear area (NA), cytoplasmic area (CA), and NA:CA ratio of exfoliated buccal mucosa cells in the study group (*n*=20) and control subjects (*n*=10) using image analyzer software. We found that there was a significant decrease in the mean CA in the study group. Though it is difficult to interpret the exact cause for the decreased cytoplasmic area, it may be attributed to the following reasons. First, the significant reduction in the mean cytoplasmic area could be due to dehydration caused by the inhalational drugs. Ryberg et al. observed that the secretion rates of whole and parotid saliva decreased by 26% and 36%, respectively, in asthmatics on medication when compared to the non-asthmatic control group.^3^ This could lead to intracellular and extracellular dehydration in oral epithelial cells, which results in a decreased mean cytoplasmic area. Similar findings in diabetic patients had been reported by Gopal et al., who hypothesized that the cytoplasmic area had reduced due to dehydration.^4^ Similarly, dehydration can also be seen in smokers, chronic alcoholics, anemic patients, and patients under radiation therapy. Seifi et al. noticed a decrease in the mean cytoplasmic area in the epithelial cells in smokers and water pipe users.^5^ They suggested that this could be due to dehydration, which is a kind of cell adaptation in response to the decrease in fluids.

The second reason for decreased cytoplasmic area could be due to an increased level of fermentable carbohydrates in the oral cavity, which is present in the inhalational drugs. According to Thomas et al. the higher rate of dental caries in asthmatics could be attributed to the fermentable carbohydrates present in the asthmatic medications.^1^ These fermentable carbohydrates do not diffuse easily into the pores of the cell membrane of oral epithelial cells, thereby leading to increased extracellular osmotic pressure. This increase in osmotic pressure causes osmotic transfer of water out of the cells, which again leads to reduction in cytoplasmic area.^4^

Thirdly, the decreased cytoplasmic area might also be a result of mucosal atrophy due to the inhalational drugs. According to Prasad et al. when smears from the atrophic oral mucosa are made, the primary pattern encompasses non-keratinized cells of the parabasal layers which are smaller but have a relatively larger nucleus, thus giving an impression of decrease in cytoplasmic area.^6^

In the present study, there was a significant increase in nuclear: cytoplasmic area ratio in the study group. It was calculated using a simple mathematical formula. The increase in NA:CA was probably due to a significant reduction in the mean cytoplasmic area in the study group, with no apparent change in the mean nuclear area.

## CONCLUSIONS

Although the findings of our study suggest that inhalational drugs do not cause any major cytomorphometric changes in the buccal mucosa, the origin of the observed changes remains uncertain. It therefore becomes necessary to assess whether these oral epithelial cells are directly affected by the drugs administered in inhalers. To the best of our knowledge, our study is the first in the English-language literature where cytomorphometric analysis was carried out in buccal mucosal cells of asthmatic patients under inhalational therapy.

## Abbreviations

CA: cytoplasmic area
NA: nuclear area
